# The Use of Public Data from Low-Cost Sensors for the Geospatial Analysis of Air Pollution from Solid Fuel Heating during the COVID-19 Pandemic Spring Period in Krakow, Poland

**DOI:** 10.3390/s21155208

**Published:** 2021-07-31

**Authors:** Tomasz Danek, Mateusz Zaręba

**Affiliations:** Department of Geoinformatics and Applied Computer Science, Faculty of Geology, Geophysics and Environmental Protection, AGH University of Science and Technology, 30-059 Krakow, Poland; tdanek@agh.edu.pl

**Keywords:** air pollution measurements, air quality monitoring, LCS, particulate matter, air quality in Krakow, anthropogenic emission, spatio-temporal geostatistics, fossil fuels

## Abstract

In this paper, we present a detailed analysis of the public data provided by low-cost sensors (LCS), which were used for spatial and temporal studies of air quality in Krakow. A PM (particulate matter) dataset was obtained in spring in 2021, during which a fairly strict lockdown was in force as a result of COVID-19. Therefore, we were able to separate the effect of solid fuel heating from other sources of background pollution, mainly caused by urban transport. Moreover, we analyzed the historical data of PM2.5 from 2010 to 2019 to show the effect of grassroots efforts and pro-clean-air legislation changes in Krakow. We designed a unique workflow with a time-spatial analysis of PM1, PM2.5, and PM10, and temperature data from Airly(c) sensors located in Krakow and its surroundings. Using geostatistical methods, we showed that Krakow’s neighboring cities are the main sources of air pollution from solid fuel heating in the city. Additionally, we showed that the changes in the law in Krakow significantly reduced the PM concentration as compared to neighboring municipalities without a fossil fuel prohibition law. Moreover, our research demonstrates that informative campaigns and education are important initiating factors in order to bring about cleaner air in the future.

## 1. Introduction

Air pollution is a major problem for modern society. Research shows that PM exposure is responsible for over 7% of deaths and more than 4% of disabilities globally [1]. Health problems can be related to short-term and long-term exposure [2]. Short-term exposure can cause asthma [3], high blood pressure, myocardial infarction, and even death, as a result of damage to the respiratory or cardiovascular systems [4,5]. Long-term exposure may contribute to the development of diseases such as lung cancer [6], pneumonia [7], crescendo angina (a type of acute coronary syndrome) [8], chronic obstructive pulmonary disease [9], and it can be even responsible for low birth weight [10]. It was observed that PM exposure is also a factor for certain neurological diseases such as Parkinson’s and Alzheimer’s disease [11].

Suburbanization and the fast-growing urban population have caused an increased demand for heating. This is particularly apparent from the beginning of autumn until the end of spring in Central Europe. The geographical location of the city of Krakow (Poland), which is situated in the Vistula River valley, and the local weather conditions favor the accumulation of pollutants in the city [12]. For years, Krakow has had a bad reputation for significantly exceeding the norms for the concentration of particulate matter in the air (the 8th worst city in the European Union (EU) according to the World Health Organization (WHO) report [13]). In 2012, the organization *Krakow Smog Alert* initiated an informative campaign focused on the bad air quality in Krakow [14]. A year later, this initiative turned into a mass social movement. The citizens of Krakow organized a public protest in which they demanded legal changes and better air quality. By the end of the same year, the Małopolska Voivodeship (the highest administrative region in Poland, which is akin to a province in other countries) assembly passed a law prohibiting the use of solid fuels for heating households. As a result of an appeal against the law, it did not enter into force as planned in 2018. In 2015, the Małopolska Voivodeship assembly passed another law prohibiting the use of solid fuels in Krakow that successfully entered into force in 2019 [15]. Presently, it is prohibited to use coal, wood, or biomass in Krakow city for central heating, any systems that emit hot air, or liquid heating installations, including fireplaces, space heaters, stoves, etc. The regulations also include a ban on the use of coal, wood, and biomass for food preparation. According to the new rules, gaseous fuels such as liquefied natural gas or light heating oil are allowed.

Air monitoring in the European Union member states is regulated by the directive 2008/50/EC of the European Parliament and of the Council of 21 May 2008 on ambient air quality and cleaner air for Europe (AAQD) [16]. In the EU, air quality stations are divided into three groups: urban traffic sites (located in urbanized areas or near high-traffic zones), urban background sites (for general population measurements), and regional background sites (general rural observations). According to the European Union Commission Staff working document *SWD(2019) 427 final*, in 2017, there were 4332 monitoring stations in all member states (278 in Poland), including 3130 PM10 total sampling points per pollutant (288 in Poland) and 1543 PM2.5 total sampling points per pollutant (111 in Poland) [17]. Krakow is located in zone PL1201 and has a total population of 756,183 and an area of 327 km^2^. In zone PL1201, three automatic measurements of PM10 and PM2.5, two manual measurements for PM10, and one manual measurement for PM2.5 are available [18]. Some of Krakow’s surrounding areas are in zone PL1203, which is the zone for the whole Małopolska area (over 14,700 km^2^). In this zone, six automatic measurements and 10 manual measurements for PM10 are available, and four manual measurements for PM2.5 are available [19]. The reference measurements and instruments used in Poland are described in the following documents: PN-EN 12341 (for gravimetric measurements) and PN-EN 16450 (for automatic measurements) [20]. The current EU and Polish standards are 25 µg/m^3^ (1-year averaging period) for PM2.5 concentrations, and 50 µg/m^3^ (24-h averaging period) and 40 µg/m^3^ (1-year averaging period) for PM10 concentrations [21].

In general, the low-cost sensors (LCS) can be categorized into electrochemical sensors, photoionization detectors, optical particle counters, and optical sensors. Currently, the European Union regulation does not allow for the use of LCS for official air quality reporting. This is related to the questionable data quality from these sensors as compared with official gravimetric measurements. LCS measurements can be affected by many weather-related factors; however, in well-prepared environments and stations, they can provide a similar data quality to official government measurements [22]. In this paper, we present research regarding the use of popular, low-cost Airly optic sensors (http://airly.eu, accessed on 29 July 2021). These sensors were used in air quality studies in Niedzica (Poland) [23] and for health risk research [24]. There was another evaluation of Airly sensors in 2018 as part of the LIFE Integrated Project “*Implementation of Air Quality Plan for Małopolska Region—Małopolska in a healthy atmosphere*”. Tests were performed according to the PN-EN 12341:2014-07 and PN-EN 16450:2017-05 standards based on an agreement concluded between the Lesser Poland Voivodeship Main Inspectorate of Environmental Protection Provincial Inspectorate for Environmental Protection in Krakow, AGH University of Science and Technology, and the community of Dobczyce, in cooperation with the Krakow Smog Alert Association. The results obtained for seven different LCS pairs of sensors (each pair produced by a different company) showed that only Airly devices provided adequate results. After calibration, the sensors provided satisfactory results in relation to the reference stations [25]. The measurement quality of the Airly sensors was also examined by AIRLAB during the Microsensors Challenge 2019, using the SET method [26]; their accuracy was 7.6 out of 10 [27]. Airly sensors were calibrated in the laboratory before the study. Measurements from a sensor in a particular location were assigned a dynamic calibration factor (created by machine learning (ML) and artificial intelligence (AI) algorithms) based on the characteristics of the location. Data are available to the public after ML/AI corrections.

Grassroots movements and local authority activities allowed for a significant improvement in air quality in Krakow [28]; however, air pollution problems still occur during spring and autumn. In this paper, we investigate whether the remaining problem seems to be mainly associated with the transport of pollutants from neighboring villages and towns. The study presented in this paper used popular, low-cost Airly sensors (for more details see http://airly.eu, accessed on 29 July 2021) to analyze whether there was a transmission of pollutants from neighboring areas to the city of Krakow in spring 2021. The impact of air pollution on human health (2005–2020) in the city of Krakow is well described by Bokwa [29] and by Traczyk and Gruszecka-Kosowska [12]. Our research focuses on the spatial-temporal analysis of the PM1, PM2.5, and PM10 contents obtained from approximately 100 Airly sensors (data available from: https://map.airly.org/ [30], accessed on 29 July 2021) located in Krakow and its surroundings. As a result of the COVID-19 pandemic, it was possible to study the effect of heating with the use of solid fuel more precisely, because of the limited traffic as compared to regular years. Research carried out by the Department of the City Traffic Engineer shows that the traffic volume during the coronavirus pandemic (at the 15 main city intersections) was as much as 40% lower as compared to pre-pandemic years [31]. We analyzed the changes in pollution levels in the city center over the last 10 years and during spring in 2021 in the city and its surroundings.

Our goals were to study:

1. The temporal and spatial distribution of air pollution in Krakow and nearby areas. We wanted to check whether pollution from heating households with fossil fuels in neighboring towns and villages migrates to Krakow and increases the level of pollution in the city. Our goal was to identify the main sources of pollution in the vicinity of Krakow and to assess the scale of the problem;
2. The effectiveness of the anti-smog policy that was gradually introduced in Krakow. Local authorities and organizations have been working since 2011 to change the air quality in Krakow. We wanted to study if these activities are related to changes in the PM concentration over the years.

The 2021 spring period was exceptionally favorable for this type of research (temporal and spatial distribution of PM). Overall, it was a period of abnormally low temperatures for this time of year, mainly due to the polar vortex disturbances. The simultaneous influx of cold Arctic air and the relatively large insolation caused successive periods of warmer temperatures and significant rapid cooling. In warmer periods, the air was cleaned of the so-called low-emission pollution, which is the main source of smog in and around Krakow [32]. With rapid cooling, there was an abrupt increase in low emissions from poor-quality heating systems often found outside the urban area, in a region not covered by the pro-clean-air legislation. This significantly helped the identification of local sources of pollution, which, in conditions of long-term, constant pollution of the environment in winter, could be hidden by the high background level.

## 2. Materials and Methods

**Figure 1:**
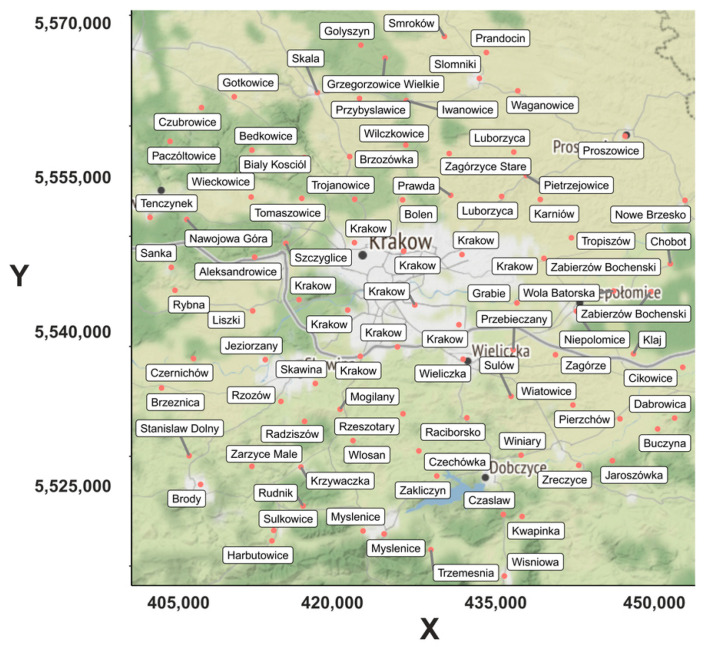
Locations of the Airly sensors used in the research (red dots) with the names of cities, towns, and villages in which they are located.

Measurements of PM1, PM2.5, and PM10 from 90 optical Airly PM Sensors were used. Figure 1 shows the locations of the sensors used in this research. The receivers were selected to cover both the city of Krakow and its surroundings. Airly sensors are one of the most popular in the Krakow area and can provide a high density of spatial data [33]. As a result of their convenience and relatively low price, they are also gaining popularity throughout the country [23,24] and around the world [34]. Airly sensors for PM measurements are optical sensors that measure light scattering. The accuracy of the results from such devices is strongly related to the physical properties of the particles, which can vary depending on location and season [35]. The manufacturer of Airly sensor states that the PM1 measurement range is 0–500 µg/m^3^ (5 µg/m^3^ accuracy in the range 0–100 µg/m^3^ and 10 µg/m^3^ in the range 101–500 µg/m^3^), and the PM2.5 and PM10 measurement range is 0–1000 µg/m^3^ (10 µg/m^3^ accuracy in the range 0–100 µg/m^3^, 10% in the range 101–500 µg/m^3^, and 20% in the range 501–1000 µg/m^3^). Aside from PM concentrations, the basic Airly sensor measures pressure in hPa (in the range 700–1200 hPa, with an accuracy of 1 hPa), the temperature in °C (in the range −40–80 °C, with an accuracy of 0.5 °C), and the humidity (in the range 0–100%, with an accuracy of 3%). The smallest measurement interval is 5 minutes. The samples are sent to the database via GSM protocol and are available from the Airly website or API. Sensors can use a solar power supply [36].

PM2.5 concentration data from the last 10 years (2010–2019) were used to determine trends using the Seasonal and Trend Decomposition with Loess method (STL), which was introduced by Cleveland et al. [37]. Raw data are difficult to interpret directly as, on certain days, there may be abnormally high PM concentration values. Unprocessed identification of such data does not necessarily translate into the general trend. Standard trend estimation methods (linear, polynomial, running average, etc.) can lead to incorrect conclusions as a result of not including seasonality or nonlinear relations. STL allows for data decomposition into trend, seasonal cycle, and even reminder component, which includes unusual data observations. The use of targeted data processing techniques can significantly improve data interpretation [38,39]. Data came from the Polish Chief Inspectorate For Environmental Protection database [40]. These data came from one to three sensors located in the city center. In the years in which more than one sensor was available, the results were averaged; the data for days in which no reading was available were recovered by linear interpolation. The major law changes were highlighted, beginning in 2012, when Krakow Smog Alert initiated an informative campaign. In 2013, grassroots civil demonstrations and protests occurred. This was the beginning of various similar events in Krakow. Thanks to public involvement and the actions of the authorities, it was possible to enforce a law prohibiting the use of solid fuels for heating. Unfortunately, the law was successfully challenged. Thanks to Environmental Law changes in Poland in 2015, it was possible to pass a second law prohibiting the use of solid fuels in Krakow in 2016 [41], which, after the adaptation period, went fully into force in 2019. During this period, various other changes were conducted to Polish regulations, including changing the emission norms in 2018. Aside from changes to the law and the education process, other factors must be considered, such as the anomalously warm winter of 2014/2015.

Despite the ban on the use of solid fuels in Krakow, a periodic increase in air pollution could be observed in the city, especially during late autumn, winter, and early spring. This situation was even observed during the COVID-19 pandemic, during which traffic was almost 40% lower than before. Thanks to the dense distribution of LCS Airly sensors, it was possible to analyze data from Krakow and its surroundings. Airly sensors located up to 30 kilometers from the center of Krakow were used for these tests. There are about 364 such sensors in the analyzed area, but they are distributed very irregularly. In certain areas, sensors are a matter of meters apart, in others, there is not a single sensor for several kilometers. To enable the correct distribution, efforts were made to select their location so that they would comprise a quasi-regular measurement grid. It is difficult to obtain a regular sensor grid using Airly API. This is because anyone can buy an Airly sensor, making their distribution irregular. The area of study (Figure 1) was divided into a regular, 100-point grid, according to the X and Y axes. In this area, 364 LCSs were available. We designed the algorithm to search for the sensor located closest to each of the 100 regularly distributed points in this area. For further analysis, we used no more than one sensor per point. The algorithm was written in R as follows:

1. Define the area of investigation;
2. Use function *makegrid* and divide into 100 regularly distributed points;
3. Read all sensors’ geographical positions;
4. Use *k-nearest-neighbours* to find the distance from sensors to a particular grid point;
5. Assign unique index number to sensors in relation to distance to grid points;
6. Choose the sensor closest to the grid point;
7. Exclude the sensors assigned to a particular grid point if the distance between them is less than ¼ of the distance between the neighboring grid points;
8. Save assigned sensor index numbers, and X and Y positions.

A satisfactory network of 90 sensors was, thus, obtained. We obtained an even distribution of measurement points, which we were able to fit into the open Airly license. To check if the Airly sensors measurements were sufficiently legible for use in the analysis, we compared them with the readings of the closest government sensors. We averaged the Airly measurements in 24-h periods and compared them with analogical scale measurements from government stations. Then, we calculated the differences between the government and Airly sensor measurements.

The general analysis of smog conditions was carried out using charts and maps created in R by fitting a thin plate spline surface to our data. This method is a special case of kriging; however, it guarantees a “smooth” surface [42], which is desirable when analyzing the dispersion propagation of pollutants. This step was performed for the identification of particularly interesting spatial-time snapshots (see the animation in the Supplementary Materials). Thereafter, we took a closer look at these data, comparing them with the temperature data.

To observe the inflow and outflow of air pollution in Krakow, the analysis of temperature changes in relation to the feeling of relative cold was performed. To observe the effect of household heating when the temperature drops below the comfort zone, KDE plots of PM were prepared. For two chosen dates, maps of PM1, PM2.5, and PM10 together with temperature were constructed. For grid preparation, the minimal curvature method was used, with grid spacing of approximately 500 meters in the X and Y directions.

## 3. Results

### 3.1. Historical Data Analysis Using Official Government Data from Reference Stations

**Figure 2:**
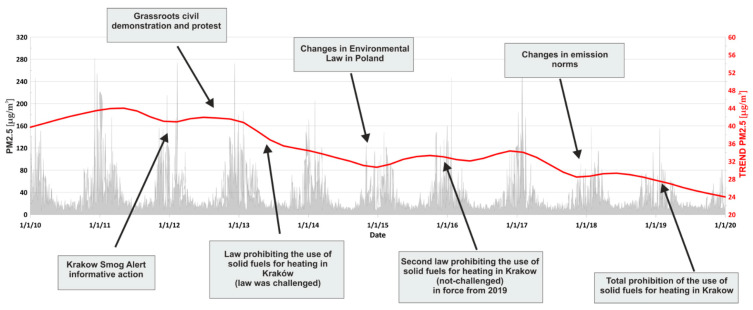
Krakow’s PM2.5 concentration (grey) and its STL trend (red) in the years 2010–2019 together with major law changes and grassroots action.

The STL decomposition of PM2.5 obtained from official government sensors is shown in Figure 2. It is clearly visible that education, grassroots initiatives, and multilevel political involvement is crucial. Along with the increase in environmental awareness in society over the years, changes in the law and a stable decrease in emissions were visible. However, selective bans on the use of solid fuels in individual cities, without taking into account surrounding towns and cities, may cause a temporary excessive increase in the concentration of PM in the city, where the prohibition law is in force.

### 3.2. Low-Cost Sensors for Inflow and Outflow Monitoring of PM1, PM2.5, and PM10 

Table 1 shows a summary of the basic statistical analysis for all data. The data were characterized by a compact distribution, i.e., outliers were rare. The mean value of PM10 for this period (7 March to 16 April 2021) was lower than the EU standard (1-year averaged—40 µg/m^3^). PM2.5 was slightly higher than that proposed by the EU standard (3-year averaged—25 µg/m^3^); however, the median for PM2.5 was approximately the same as the standard. 

For a better understanding of the air pollution problem in Krakow, an analysis of 960 h was conducted. Table 2 shows the correlation coefficients between all measured parameters for 90 detectors, for all time points. It was clear to see that PM1, PM2.5, and PM10 were correlated. 

**Figure 3:**
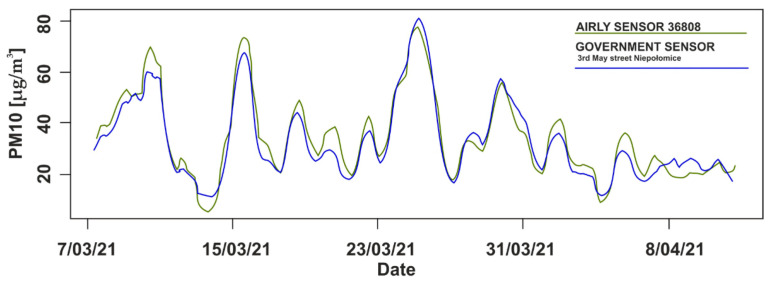
Twenty-four-hour averaged measurements of PM10 from government stations (blue) and the Airly sensor (green) in Niepołomice.

From all 90 sensors, sensor 36808 in Niepołomice was the closest to a government sensor. The government station is located on 3 May Street in Niepołomice (SE part of the investigated area). Table 3 shows statistics for the calculated time-series difference between 24-h averaged measurements of PM10 from government stations (GOV) and the Airly sensor in Niepołomice. The correlation coefficient for these measurements between 7 March and 16 April 2021 was 0.93. A comparison between the 24-h averaged measurements of Airly sensor 36808 and the government sensor located on 3 May street is shown in Figure 3.

### 3.3. Analysis of Air Pollution Inflow and Outflow According to Temperature Changes

**Figure 4:**
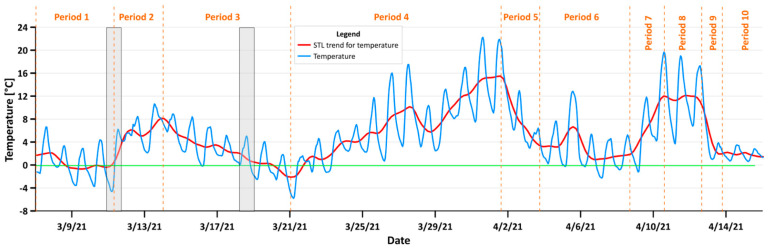
The temperature in Krakow between the 7th of March and 16th April (blue) and the temperature STL trend (red). The green line indicates zero Celsius degree point; grey boxes are days chosen for the inflow and outflow study. Orange marks represent 10 different temperature periods.

**Figure 5:**
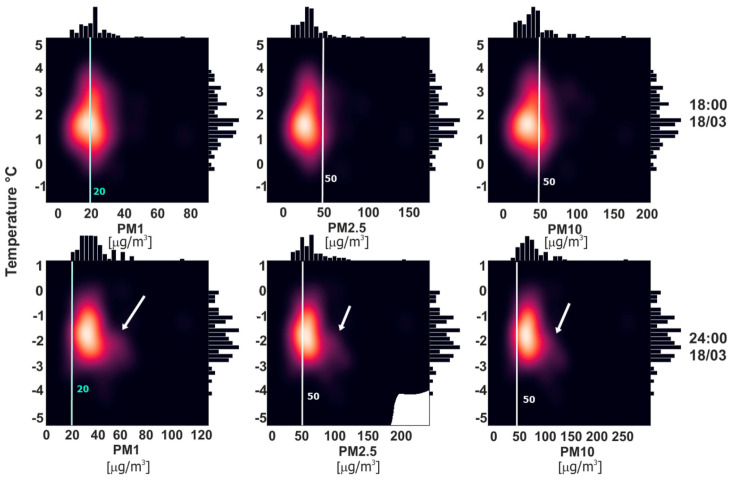
Kernel density estimate plots of PM1, PM2.5, PM10 and temperature at 18:00 and 24:00 on the 18th of March. Arrows indicate the increase in PM related to temperature and also to PT and PMV.

Figure 4 shows the temperature measurements from 7 March to 16 April 2021 together with the STL trend. Our aim was to show the inflow and outflow of air pollution in Krakow. To indicate the proper time and date, it was necessary to deeply study the temperature changes, as this is one of the main factors affecting whether people heat their households or not. It was possible to designate 10 temperature intervals: the first was the 7 to 10 March, which was stable at around 0 °C; the second was from the 11 to 14 March, during which the average temperature trend increase was visible; the next was a slowly decreasing period from 14 to 21 March, at the end of which the temperature reached its minimum value; the 4th period was up until the 2 April, when temperature stably increased (excepting a 1-day trend break); the next period was from the 2 to 5 April, wherein the temperature decreased to around 1–2 °C and stabilized on this value for 5 days; then, there were four three-day periods, in which the temperature first rose, then stabilized, before dropping again, and stabilizing once more in the last period. The 11th of March was chosen to study the effect of the air pollution outflow, and the 18th of March was chosen to study the effect of the air pollution inflow. Firstly, those days were characterized by an upward or downward temperature trend. Secondly, according to the relationship between the Predicted Mean Vote (PMV) and Perceived Temperature (PT) indicator in the study by Jendritzky et al. [43], it was shown that most people classified temperature between 0 and 20 °C as comfortable, and when the temperature decreases below 0 °C, most people classify it as cold. Figure 5 shows the increase in PM concentrations when the temperature dropped below the aforementioned comfortable temperature. In the first-row kernel density estimate plots for PM1, PM2.5, and PM10 versus temperature from the 18th of March, 18:00 was plotted. The second row contains the same plots but from 24:00. At 18:00, the temperature was above zero, and for all temperature points, the distribution of PM was similar. At 24:00, the distortion in the distribution symmetry is visible. White arrows indicate the increase in PM in the air. This is in clear accordance with the PMV and PT study.

For the air pollution inflow, measurements from 12:00, 18:00, and 24:00 were used. For the air pollution outflow, measurements from 00:00, 04:00, and 08:00 were used. This is because people begin to heat their houses after sunset, which, in March, is around 17:00. To observe the effect of inflow, we analyzed midday, when the PM concentration was expected to be low at almost every sensor, then at 18:00, when the PM should have been visible in the surroundings of Krakow, and then at midnight, when the inflow effect was expected to be visible in the city. For outflow, we began at midnight, when the concentration of PM in the city due to inflow was expected to be high. We then proceeded in 4-h intervals to investigate the change.

**Figure 6:**
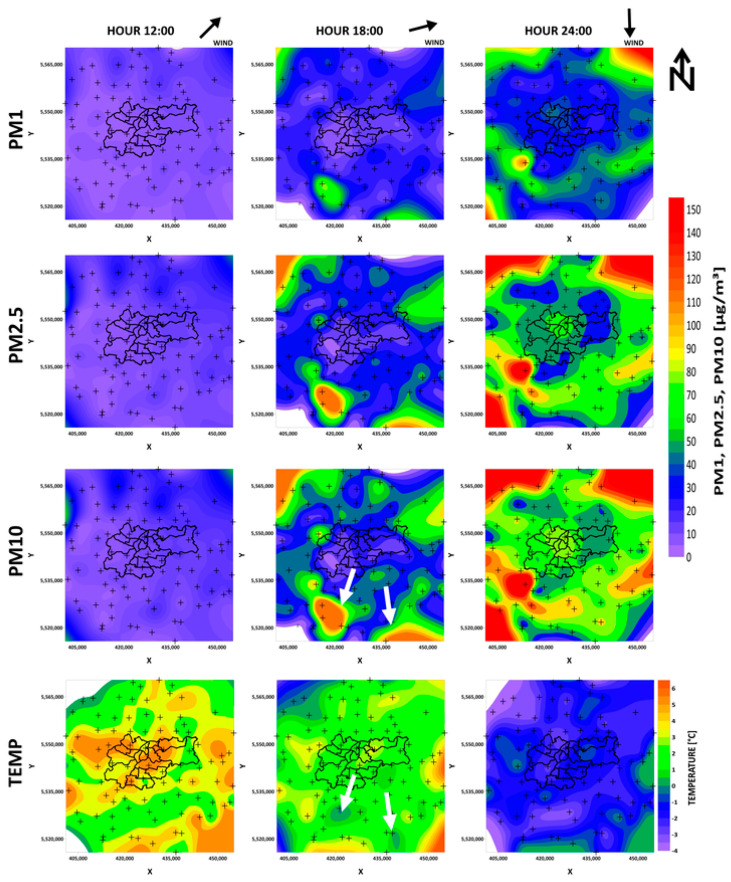
Temperature maps of air pollutions entering (PM1, PM2.5, PM10) Krakow on 18 March 2020. White arrows indicate areas in which the temperature was below the comfort zone.

**Figure 7:**
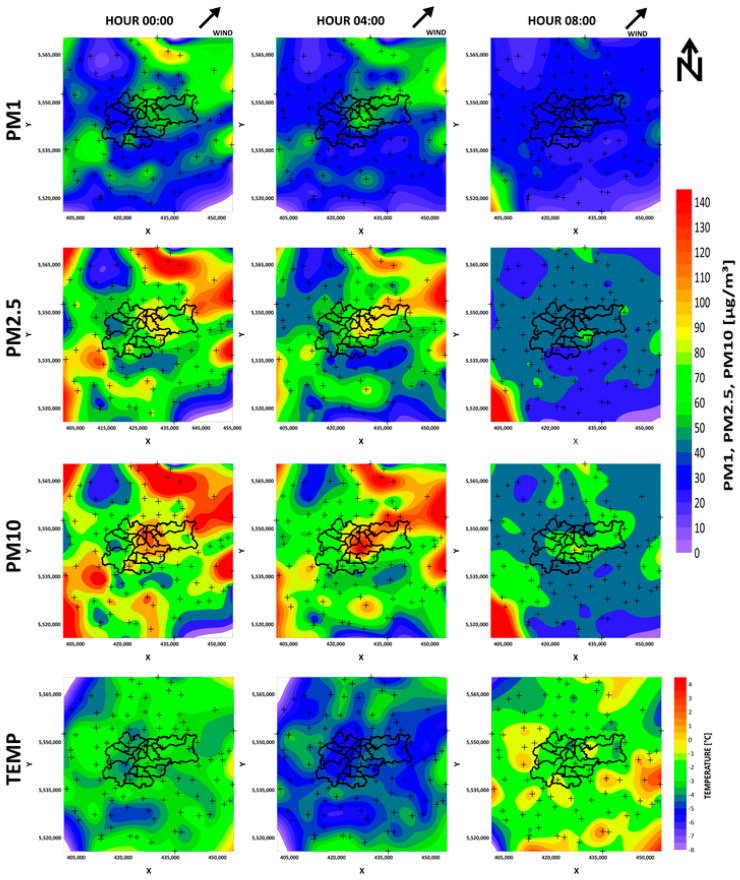
Temperature maps of air pollution leaving (PM1, PM2.5, PM10) Krakow on 11 March 2020.

Figure 6 shows the air pollution inflow to Krakow, while Figure 7 shows the air pollution outflow. In the first row, the PM1 concentration was presented for three different hours, the second presents similar maps for PM2.5, the third shows PM10, and the fourth presents the temperature. Crosses represent LCS. Considering the inflow of air pollutants into Krakow from surrounding towns and cities, it is clear to see that, at noon, Krakow city was the warmest area on the map; however, the PM concentrations were low in and around the city. At 18:00, the temperature was stable; this was similar at every point with an average of approximately 1 °C. In certain places outside Krakow, air pollution was starting to increase. These were places where the temperature was lower than in the surrounding receivers, close to the thermal comfort limit (see white arrows in Figure 6). However, despite a significant increase in PM in neighboring towns and villages, the air quality in Krakow remained good. This situation changed with time. At midnight, it could be clearly seen that the pollutants diffused into Krakow; however, the level of PM contamination was still lower in the city of Krakow. The maps show that PM10 pollution increased the most, and PM1 pollution the least.

In the case of an outflow of pollutants, it is again visible in the concentration analysis that, at midnight, the accumulation of pollutants was the result of their diffusion from neighboring municipalities. The 11th of March was chosen because, on this day, the temperature in the morning began to rise above the comfort temperature. In this way, we were able to exclude the additional influx of pollutants resulting from heating houses in the morning.

## 4. Discussion

The statistics in Table 1 demonstrate that the distribution of Airly data from March to April 2021 was compact. There were no outliers and abnormally high or low indications. This was probably related to the Airly data preparation, as their measurements are filtered and corrected by their internal machine-learning and artificial intelligence algorithms. According to the correlation coefficients values in Table 2, it is clearly visible that PM1, PM2.5, and PM10 were correlated. This was expected as the individual particulate matter of a given fraction also contains information about particles of a smaller fraction. In this case, the PM2.5 data were used to prepare an animation (see the Supplementary Materials) to show the hourly changes in spring 2021. It was, therefore, also justified to use the PM2.5 historical data from the government stations to show the general trend of air pollution contamination in Krakow over the last decade. 

Airly sensor 36808 in Niepołomice provided comparable results (averaged over a 24-h period) to the closest government station. They were highly correlated, i.e., the correlation coefficient was 0.93 for the investigated period and the average difference between their measurements was 3 µg/m^3^. There were days (see Figure 3) in which the Airly sensors inflated or underestimated the results; however, the general measurement distribution was similar to that provided by the government sensors. This was sufficient to trace relative spatial-temporal changes with Airly LCS measurements. Aside from the comparative analysis of indications from government stations and Airly sensors, research conducted by the Marshal’s Office in the Małopolska Region showed that Airly sensors give reliable measurements [25], better than other tested devices. Airly provides results that are already processed and corrected with the use of their machine-learning algorithms. However, LCS have their limitations and can easily be affected by various external factors. It can be difficult to correct raw data, while taking into account all factors. On the other hand, official government stations did not provide sufficient spatial coverage for our study. The presented accuracy of Airly sensors was sufficient to track the relative changes in air pollution in Krakow. The potential anomalous indication of a single station was easy to eliminate by analyzing the distribution. We did not notice such indications on our maps.

To analyze the inflow (Figure 6) and outflow (Figure 7) of air pollution in Krakow, we chose days in which the temperature changed from comfortable to cold (inflow), and from cold to comfortable. It was practical to find periods in which the temperature changed from cold to comfortable or vice versa. Whether people start to heat their homes basically depends on their perception of the temperature, so there was no reason to choose the 28th of March for example, which was characterized by a break in the upward trend, as even if the temperature dropped, it was still perceived as comfortable. This effect is clearly visible in Figure 5, which shows the kernel density estimate plots for different PM for different hours in relation to temperature. All PM concentrations rose in areas where the temperature dropped below 0 °C. This effect can also be observed in the maps. Air pollution started to increase in certain places outside Krakow at around 18:00. On the temperature maps, there were places where the temperature was lower than in the surrounding receivers. Aside from the relatively low value, the temperature in these places was very close to the thermal comfort limit (see Figure 6 white arrows). This observation could be very important in the future for forecasting air pollution.

Figure 6 and Figure 7 contain wind arrow indicators; however, the wind speed was low on these days. The maps presented in Figure 6 and Figure 7, and in the Supplementary animation, demonstrate that the process responsible for air pollution arriving to the city was related to both pollution blowing in on the wind and particular matter from surrounding towns and villages in the hills around diffusing and settling in Krakow, which is located in the valley. This valuable observation will allow for the better modeling of pollutant transport in the future.

PM contamination increased in Krakow depending on the fraction size. The highest increase could be seen for PM10, and the lowest for PM1. This may be because the lighter fractions could more easily remain at high altitudes and were not measured by devices located at a height of 1.5–8 m. The other reason may be related to the sources of air pollution. We considered fossil fuel use for household heating, so higher PM10 contamination was expected. In the case of pollution outflow, the city was most exposed to heavy PM10 dust, which, due to its size, is present at highest concentrations in the morning. The city of Krakow, as a result of its location in the valley, does not have favorable conditions for the outflow of pollutants, which was clearly shown by the distributions on the maps. We can see in Figure 7 that PM10 contamination remained in the city the longest and higher concentrations coincided with the course of rivers. Rivers tend to have an erosive base and usually flow at their lowest point. Perhaps it is because of their location that air pollution stays in these areas the longest. Another reason may be the increased presence of water vapor and the formation of mists in those areas. This may act to impede air movement and, consequently, the migration of heavier fractions of air pollution.

## 5. Conclusions

Our research clearly showed that a dense network of Airly low-cost sensors aids in the spatial and time analyses of air pollution inflow and outflow in Krakow. The ongoing COVID-19 pandemic allowed us to analyze the effect of pollutant diffusion from neighboring municipalities to Krakow, without introducing noise resulting from car traffic. It was shown that, apart from the daily temperature changes themselves, perceptible thermal comfort is an important factor. The subjective feeling of cold influences whether the inhabitants of neighboring municipalities heat their houses or not.

We utilized the STL decomposition method, the 10-year trend of PM2.5 concentration, the PM2.5 concentration, and data concerning the law and educational changes. Aside from physical factors such as the warm winter in 2014/2015, it was demonstrated that the Krakow and Małopolska Voivodeship authorities performed effective steps to improve air quality in the city. Moreover, it was demonstrated that the education and engagement of the local community were effective and important in this regard. A downward trend was visible from the time when the Krakow Smog Alert began its informative campaign in 2012. 

The law prohibiting the use of solid fuels for heating in Krakow city brought about the intended reduction in PM. Unfortunately, as a result of its geographic location and the lack of similar bans in neighboring municipalities, Krakow is still exposed to pollution that exceeds air quality standards. Of course, long-term low emissions and its downwind transport completely fill urban areas over time; however, in the transition seasons, in which increased emissions last several days, the air in the city was of radically better quality than in the outskirts.

Our study clearly showed that the influx of PM1, PM2.5, and PM10 by diffusion was the greatest from the following towns: from the west—Rybna, Czernichów, and Brzeźnica; from the southwest—Brody, Skawina, Rzozów, Radziszów, Krzywaczka, Czechówka, and Zakliczyn; from the southeast—Dobczyce, Czasław, Kwapinka, Wieliczka, and Niepołomice; from the northeast—Proszowice, Waganowice, Słomniki, and Prandocin; from the northwest—Więckowice, Paczółtowice, Czubrowice, Gotkowice, Skała, and Gołyszyn. It also seems that the PM may be transported from sources a greater distance to the south, perhaps from Podhale, but this will be the focus of future research. The longest-lasting deposited dust was that of the PM10 fraction.

## Figures and Tables

**Table 1:** Summary of basic statistics for the measured parameters from Airly sensors in spring in Krakow, 2021, during the COVID-19 pandemic.

	Temp (°C)	Pressure (hPa)	Humidity (%)	PM1	PM2.5	PM10
				µg/m^3^		
**Min**	−7.37	994.40	17.65	0.02	0.21	0.28
**1st Qu**	1.02	1012.80	63.47	9.62	14.69	17.21
**Median**	3.72	1017.50	76.19	16.00	25.14	31.49
**Mean**	4.81	1016.80	74.39	18.35	29.72	37.12
**3rd Qu**	7.71	1021.10	87.25	23.36	37.68	49.56
**Max**	28.46	1032.70	100.00	148.54	305.25	376.18

**Table 2:** Correlation coefficients for the measured parameters from Airly sensors for all data points.

		Temp (°C)	Pressure (hPa)	Humidity (%)	PM1	PM2.5	PM10
					µg/m^3^		
**Temp (°C)**		1.000	0.048	−0.583	−0.369	−0.357	−0.374
**Pressure (hPa)**		0.048	1.000	0.111	0.241	0.258	0.235
**Humidity (%)**		−0.583	0.111	1.000	0.342	0.341	0.354
**PM2.5**	**µg/m^3^**	−0.369	0.241	0.342	1.000	0.997	0.997
**PM1**		−0.357	0.258	0.341	0.997	1.000	0.995
**PM10**		−0.374	0.235	0.354	0.997	0.995	1.000

**Table 3:** Statistics for the difference in PM10 measurements between government station and Airly sensor 36808 for 24-h averaged periods between 7 March and 16 April 2021.

	Min	1st Qu	Median	Mean	3rd Qu	Max
**GOV and 36808 sensors difference (µg/m^3^)**	−19.157	−7.425	−3.593	−3.001	1.069	17.407

## Data Availability

1. Publicly available datasets from Airly sensors were analyzed in this study. This data can be found here: (https://map.airly.org/, accessed on 29 July 2021). Airly API documentation is available here: (https://developer.airly.org/en/docs, accessed on 29 July 2021). 2. Publicly available datasets from the Chief Inspectorate For Environmental Protection database were analyzed in this study. This data can be found here: (http://powietrze.gios.gov.pl/pjp/home, accessed on 29 July 2021). API documentation is available here: (http://powietrze.gios.gov.pl/pjp/content/api, accessed on 29 July 2021).

## Supplementary Materials

Animation is available online at https://www.mdpi.com/article/10.3390/s21155208/s1, Animation S1: PM2.5 changes in Krakow and its neighborhood between 7 March and 16 April 2021.
